# Profibrotic, Electrical, and Calcium-Handling Remodeling of the Atria in Heart Failure Patients With and Without Atrial Fibrillation

**DOI:** 10.3389/fphys.2018.01383

**Published:** 2018-10-09

**Authors:** Cristina E. Molina, Issam H. Abu-Taha, Qiongling Wang, Elena Roselló-Díez, Marcus Kamler, Stanley Nattel, Ursula Ravens, Xander H. T. Wehrens, Leif Hove-Madsen, Jordi Heijman, Dobromir Dobrev

**Affiliations:** ^1^Institute of Pharmacology, West German Heart and Vascular Center, University Duisburg-Essen, Essen, Germany; ^2^Biomedical Research Institute Barcelona (IIBB-CSIC) and Biomedical Research Institute Sant Pau, Hospital de Sant Pau, Barcelona, Spain; ^3^Institute of Experimental Cardiovascular Research, University Medical Center Hamburg-Eppendorf, Hamburg, Germany; ^4^Cardiovascular Research Institute – Department of Molecular Physiology and Biophysics, Baylor College of Medicine, Houston, TX, United States; ^5^Cardiac Surgery Department, Hospital de la Santa Creu i Sant Pau, Barcelona, Spain; ^6^Department of Thoracic and Cardiovascular Surgery, West German Heart and Vascular Center Essen, University Hospital Essen, Essen, Germany; ^7^Department of Medicine, Montreal Heart Institute and Université de Montréal, Montreal, QC, Canada; ^8^Department of Pharmacology and Therapeutics, McGill University, Montreal, QC, Canada; ^9^Institute of Experimental Cardiovascular Medicine, University Heart Center Freiburg, University of Freiburg, Bad Krozingen, Germany; ^10^Institute of Physiology, Medical Faculty Carl Gustav Carus, TU Dresden, Dresden, Germany; ^11^Department of Cardiology, CARIM School for Cardiovascular Diseases, Maastricht University, Maastricht, Netherlands

**Keywords:** atrial fibrillation, heart failure, heart failure with reduced ejection fraction, human atrial cardiomyocytes, Ca^2+^ handling

## Abstract

Atrial fibrillation (AF) and heart failure (HF) are common cardiovascular diseases that often co-exist. Animal models have suggested complex AF-promoting atrial structural, electrical, and Ca^2+^-handling remodeling in the setting of HF, but data in human samples are scarce, particularly regarding Ca^2+^-handling remodeling. Here, we evaluated atrial remodeling in patients with severe left ventricular (LV) dysfunction (HFrEF), long-standing persistent (‘chronic’) AF (cAF) or both (HFrEF-cAF), and sinus rhythm controls with normal LV function (Ctl) using western blot in right-atrial tissue, sharp-electrode action potential (AP) measurements in atrial trabeculae and voltage-clamp experiments in isolated right-atrial cardiomyocytes. Compared to Ctl, expression of profibrotic markers (collagen-1a, fibronectin, periostin) was higher in HFrEF and HFrEF-cAF patients, indicative of structural remodeling. Connexin-43 expression was reduced in HFrEF patients, but not HFrEF-cAF patients. AP characteristics were unchanged in HFrEF, but showed classical indices of electrical remodeling in cAF and HFrEF-cAF (prolonged AP duration at 20% and shorter AP duration at 50% and 90% repolarization). L-type Ca^2+^ current (I_Ca,L_) was significantly reduced in HFrEF, cAF and HFrEF-cAF, without changes in voltage-dependence. Potentially proarrhythmic spontaneous transient-inward currents were significantly more frequent in HFrEF and HFrEF-cAF compared to Ctl, likely resulting from increased sarcoplasmic reticulum (SR) Ca^2+^ load (integrated caffeine-induced current) in HFrEF and increased ryanodine-receptor (RyR2) single-channel open probability in HFrEF and HFrEF-cAF. Although expression and phosphorylation of the SR Ca^2+^-ATPase type-2a (SERCA2a) regulator phospholamban were unchanged in HFrEF and HFrEF-cAF patients, protein levels of SERCA2a were increased in HFrEF-cAF and sarcolipin expression was decreased in both HFrEF and HFrEF-cAF, likely increasing SR Ca^2+^ uptake and load. RyR2 protein levels were decreased in HFrEF and HFrEF-cAF patients, but junctin levels were higher in HFrEF and relative Ser2814-RyR2 phosphorylation levels were increased in HFrEF-cAF, both potentially contributing to the greater RyR2 open probability. These novel insights into the molecular substrate for atrial arrhythmias in HF-patients position Ca^2+^-handling abnormalities as a likely trigger of AF in HF patients, which subsequently produces electrical remodeling that promotes the maintenance of the arrhythmia. Our new findings may have important implications for the development of novel treatment options for AF in the context of HF.

## Introduction

Atrial fibrillation (AF) and heart failure (HF) are common cardiovascular diseases, negatively affecting morbidity and mortality of millions of patients worldwide (Benjamin et al., 2018). AF and HF frequently co-exist, and HF is a strong independent risk factor for the development of AF (Ling et al., 2016; Sartipy et al., 2017). AF prevalence increases with the severity of HF, ranging from 5% in NYHA Class I to 40–50% in NYHA Class IV (Andrade et al., 2014). The presence of AF in HF patients is associated with a worse prognosis (Wang et al., 2003; Mogensen et al., 2017). In agreement, the CASTLE-AF study has recently demonstrated that maintenance of sinus rhythm in HF patients with reduced left ventricular (LV) ejection fraction (HFrEF) through catheter ablation of AF significantly reduces mortality (Marrouche et al., 2018).

The common clinical coincidence of AF and HF may in part be due to their shared dependence on risk factors such as hypertension and diabetes. In addition, several bidirectional mechanistic interactions between AF and HF may promote the co-existence of both diseases in patients (Heijman et al., 2013; Hohendanner et al., 2018). For example, the atrial contribution to cardiac output is lost in the setting of AF and AF may promote ventricular dysfunction when ventricular rate is not well controlled. On the other hand, HF chronically elevates atrial pressure and causes atrial stretch, while activating systemic neurohumoral signaling pathways that affect the atria. Both components are expected to produce AF-promoting atrial remodeling. Indeed, AF is increasingly regarded as a symptom of an atrial cardiomyopathy produced by a wide variety of pathophysiological processes (Goette et al., 2017), which might be the first clinical sign of compromised ventricular function.

Animal models have identified complex atrial electrical remodeling in HF, which differs depending on species, as well as type (myocardial infarction vs. ventricular tachypacing) and duration of HF (Pandit and Workman, 2016). By contrast, structural remodeling, including activation of profibrotic signaling pathways, is a consistent finding in most HF models (Li et al., 1999; Cardin et al., 2003; Milliez et al., 2005; Yamada et al., 2017). Finally, accumulating evidence also suggests an important role for Ca^2+^-handling abnormalities in the increased susceptibility to AF in various HF models (Yeh et al., 2008; Lugenbiel et al., 2015; Pluteanu et al., 2018).

Multiple components of atrial remodeling in patients have been characterized for different forms of AF (Brundel et al., 2002; Schotten et al., 2011; Jalife and Kaur, 2015; Simon et al., 2016). Electrical remodeling (notably shortening of action potential [AP] duration [APD]) (Dobrev et al., 2001; Voigt et al., 2012; Schmidt et al., 2015) and structural remodeling (hypertrophy and increased fibrosis) are consistent findings in patients with long-standing persistent (‘chronic’) AF (cAF) (Polyakova et al., 2008; Cao et al., 2010). Ca^2+^-handling abnormalities have been identified in both paroxysmal AF and cAF, although the underlying molecular mechanisms are distinct (Voigt et al., 2012, 2014; Beavers et al., 2013). However, information about AF-promoting atrial remodeling in HFrEF patients is limited and inconsistent (Ling et al., 2016; Pandit and Workman, 2016), with reduced, unchanged or prolonged cardiomyocyte APD being reported in HFrEF patients (Schreieck et al., 2000; Workman et al., 2009; Schmidt et al., 2017). On electroanatomical mapping, APDs are rather prolonged in HF patients (Sanders et al., 2003), paralleling findings in animal models (Li et al., 2000). Similar to animal models, atrial structural remodeling, including fibrosis, is commonly observed in HF patients (Xu et al., 2004; Akkaya et al., 2013). However, whether HF is associated with atrial Ca^2+^-handling abnormalities in humans and whether the underlying mechanisms are similar to those observed in patients with AF is not known. Moreover, patients with HF-dependent AF are subjected to both HF- and AF-related remodeling. Due to the cross-talk between both processes, the resulting remodeling is likely different from and more complex than the sum of the individual processes (Cha et al., 2004; Heijman et al., 2013; Goette et al., 2017), but has not yet been characterized in atrial samples from patients.

Here, we assessed for the first time atrial profibrotic, electrical and Ca^2+^-handling remodeling in atrial samples from patients with HFrEF and HFrEF-cAF to sinus-rhythm patients with normal LV ejection fraction (Ctl). We obtained evidence for substantial profibrotic and Ca^2+^-handling remodeling in HFrEF patients, but did not find indices of classical electrical remodeling. Proarrhythmic diastolic Ca^2+^-release events and profibrotic remodeling were similarly observed in HFrEF-cAF patients, along with classical AF-related electrical remodeling, consistent with the more complex pathophysiology present in these patients. Overall, our new data position Ca^2+^-handling abnormalities as a potential early component of HF-related atrial remodeling, which in fibrotic atria may initiate AF episodes that induce electrical remodeling, promoting arrhythmia-stabilizing reentry and AF persistence.

## Materials and Methods

### Human Tissue Samples

Right-atrial (RA) appendages were obtained from 79 Ctl patients (LV ejection fraction > 50%), 40 patients with HFrEF, 35 patients with cAF and 36 HFrEF-cAF undergoing open-heart surgery. Mean LV ejection fraction was <35% for HFrEF and HFrEF-cAF. Patient characteristics are provided in **Tables 1–3**. Experimental protocols were approved by ethical review boards of Dresden University of Technology, Hospital de la Santa Creu i Sant Pau and University Hospital Essen, and were conducted in accordance with the Declaration of Helsinki. Each patient gave written informed consent. The tissue samples were collected just prior to atrial cannulation for extracorporeal circulatory bypass, stored in Tyrode solution and transferred to the laboratory for AP recordings in trabeculae, cardiomyocyte isolation for voltage-clamp studies, or freezing in liquid nitrogen for biochemical and biophysical experiments, as detailed below.

**Table 1:** Clinical characteristics of patient samples employed for multicellular action potential recordings.

	Ctl	HFrEF	cAF	HFrEF-cAF
Patients (*n*)	15	15	15	15
Female gender	5 (33.3%)	5 (33.3%)	3 (20.0%)	2 (13.3%)
Age (years)	67.7 ± 2.43	68.2 ± 1.31	73.1 ± 1.63	71.1 ± 1.73
BMI (kg/m^2^)	28.3 ± 0.93	27.1 ± 1.37	28.4 ± 1.26	27.5 ± 1.10
CAD	7 (46.7%)	8 (53.3%)	4 (26.7%)	2 (13.3%)
AVD/MVD	3 (20.0%)	4 (26.7%)	9 (60.0%)	9 (60.0%)
CAD + AVD/MVD	5 (33.3%)	3 (20.0%)	2 (13.3%)	4 (26.7%)
Hypertension	14 (93.3%)	13 (86.7%)	15 (100%)	14 (93.3%)
Diabetes	6 (40.0%)	7 (46.7%)	6 (40.0%)	6 (40.0%)
Dyslipidemia	11 (73.3%)	10 (66.7%)	11 (78.6%)	11 (78.6%)
LAD (mm)	44.0 ± 1.44	44.6 ± 1.58	50.38 ± 1.57^1^	54.3 ± 1.93^∗,#^
LVEF (%)	60.9 ± 1.81	27.8 ± 1.57^∗^	61.73 ± 1.85^#^	29.5 ± 1.55^∗,$^
Digitalis	0 (0.0%)	1 (6.7%)	5 (33.3%)	3 (20.0%)
ACE inhibitors	8 (53.3%)	9 (60.0%)	8 (53.3%)	11 (73.3%)
AT_1_ blockers	4 (26.7%)	7 (46.7%)	7 (46.7%)	1 (6.7%)
Beta-blockers	13 (86.7%)	14 (93.3%)	10 (66.7%)	14 (93.3%)
Calcium-antagonists	2 (13.3%)	0 (0.0%)	3 (20.0%)	0 (0.0%)
Diuretics	7 (46.7%)	11 (73.3%)	14 (93.3%)	11 (73.3%)
Nitrates	3 (20.0%)	0 (0.0%)	2 (13.3%)	2 (13.3%)
Lipid-lowering drugs	14 (93.3%)	7 (46.7%)	10 (66.7%)	9 (60.0%)

*Values are presented as mean ± SEM or number of patients (%). ACE, angiotensin-converting enzyme; AT, angiotensin receptor; AVD/MVD, aortic/mitral valve disease; BMI, body mass index; CAD, coronary artery disease; LAD, left atrial diameter; LVEF, left ventricular ejection fraction. ^∗^ indicates *P* < 0.01 vs. Ctl, ^#^ indicates *P* < 0.01 vs. HFrEF, ^$^ indicates *P* < 0.01 vs. cAF. ^1^LAD in cAF is borderline significantly increased vs. Ctl (*P* = 0.051). CAD, AVD/MVD, and CAD + AVD/MVD reflect the indications for cardiac surgery (bypass surgery, valve replacement or a combination of both).*

**Table 2:** Clinical characteristics of patient samples used for voltage-clamp recordings in isolated atrial cardiomyocytes.

	Ctl	HFrEF	cAF	HFrEF-cAF
Patients (*n*)	37	8	20	8
Female gender	22 (59.5%)	5 (62.5%)	12 (60.0%)	2 (25.0%)
Age (years)	62.6 ± 2.20	69.9 ± 4.10	69.0 ± 1.83	64.8 ± 7.20
BMI (kg/m^2^)	N/A	N/A	N/A	N/A
CAD	11 (29.7%)	4 (50.0%)	3 (15.0%)	1 (12.5%)
AVD/MVD	19 (51.4%)	2 (25.0%)	13 (65.0%)	5 (62.5%)
CAD + AVD/MVD	7 (18.9%)	2 (25.0%)	4 (20.0%)	2 (25.0%)
Hypertension	18 (48.6%)	4 (50%)	14 (70.0%)	3 (37.5%)
Diabetes	11 (29.7%)	3 (37.5%)	7 (35.0%)	1 (12.5%)
Dyslipidemia	20 (54.1%)	5 (62.5%)	9 (45.0%)	3 (37.5%)
LAD (mm)	39.5 ± 1.3	43.3 ± 5.1	52.3 ± 3.04^∗^	47.7 ± 4.2
LVEF (%)	64.5 ± 1.9	25.1 ± 2.4^∗^	52.4 ± 1.40^∗,#^	28.7 ± 4.4^∗,$^
Digitalis	1 (2.7%)	0 (0.0%)	6 (30.0%)*	4 (50.0%)*
ACE inhibitors	10 (27.0%)	3(37.5%)	8 (40.0%)	2 (25.0%)
AT_1_ blockers	8 (21.6%)	0 (0.0%)	6 (30.0%)	1 (12.5%)
Beta-blockers	13 (35.1%)	5 (62.5%)	10 (50.0%)	3 (37.5%)
Calcium-antagonists	0 (0.0%)	0 (0.0%)	0 (0.0%)	0 (0.0%)
Diuretics	8 (21.6%)	3 (37.5%)	10 (50.0%)	5 (62.5%)
Nitrates	13 (35.1%)	0 (0.0%)	7 (35.0%)	0 (0.0%)
Lipid-lowering drugs	19 (51.3%)	4 (50%)	9 (45.0%)	2 (25%)

*Values are presented as mean ± SEM or number of patients (%). ACE, angiotensin-converting enzyme; AT, angiotensin receptor; AVD/MVD, aortic/mitral valve disease; BMI, body mass index; CAD, coronary artery disease; LAD, left atrial diameter; LVEF, left ventricular ejection fraction; N/A, not available. ^∗^ indicates *P* < 0.05 vs. Ctl, ^#^ indicates *P* < 0.05 vs. HFrEF, ^$^ indicates *P* < 0.05 vs. cAF. CAD, AVD/MVD, and CAD + AVD/MVD reflect the indications for cardiac surgery (bypass surgery, valve replacement or a combination of both).*

**Table 3:** Clinical characteristics of patient samples employed for biochemical experiments.

	Ctl	HFrEF	HFrEF-cAF
Patients (*n*)	27	17	13
Female gender	11 (40.7%)	3 (17.6%)	4 (30.8%)
Age (years)	69.6 ± 2.37	63.4 ± 3.10	71.4 ± 1.51
BMI (kg/m^2^)	29.3 ± 0.91	27.9 ± 1.19	27.9 ± 2.02
CAD	3 (11.1%)	7 (46.7%)*	4 (30.8%)
AVD/MVD	19 (70.4%)	4 (26.7%)*	8 (61.5%)
CAD + AVD/MVD	5 (18.5%)	4 (26.7%)*	1 (7.7%)
Hypertension^1^	21 (77.8%)	7 (50.0%)	9 (69.2%)
Diabetes^1^	8 (30.8%)	4 (30.8%)	4 (30.8%)
Dyslipidemia^1^	14 (58.3%)	7 (50.0%)	2 (16.7%)
LAD (mm)	39.5 ± 0.57	43.9 ± 1.04^∗^	47.0 ± 1.30^∗^
LVEF (%)	62.7 ± 1.22	33.3 ± 1.09^∗^	34.2 ± 1.74^∗^
Digitalis	0 (0.0%)	0 (0.0%)	1 (7.7%)
ACE inhibitors	7 (25.9%)	10 (58.8%)	5 (38.5%)
AT_1_ blockers	5 (18.5%)	0 (0.0%)	2 (15.4%)
Beta-blockers	15 (55.6%)	16 (94.1%)*	9 (69.2%)
Calcium-antagonists	9 (33.3%)	2 (11.8%)	1 (7.7%)
Diuretics	12 (44.4%)	13 (76.5%)	7 (53.8%)
Nitrates	2 (7.4%)	0 (0.0%)	0 (0.0%)
Lipid-lowering drugs	11 (40.7%)	10 (58.8%)	4 (30.8%)

*Values are presented as mean ± SEM or number of patients (%). ACE, angiotensin-converting enzyme; AT, angiotensin receptor; AVD/MVD, aortic/mitral valve disease; BMI, body mass index; CAD, coronary artery disease; LAD, left atrial diameter; LVEF, left ventricular ejection fraction. ^∗^ indicates *P* < 0.05 vs. Ctl. ^1^Information on hypertension, diabetes and dyslipidemia was available for 27, 26, and 24 of the 27 Ctl patients, for 14, 13, and 14 of the 17 HFrEF patients, and for 13, 13, and 12 of the 13 HFrEF-cAF patients, respectively. CAD, AVD/MVD, and CAD + AVD/MVD reflect the indications for cardiac surgery (bypass surgery, valve replacement or a combination of both).*

### Western Blot Analysis

Western blot analyses were performed as previously described (El-Armouche et al., 2006; Shanmugam et al., 2011). In brief, atrial lysates were subjected to electrophoresis on 5% or 10% acrylamide gels, or 10% Bis-Tris gels (Thermo Fisher Scientific, Waltham, MA, United States) for analyses of sarcolipin, followed by transfer onto polyvinyl difluoride (PVDF) or nitrocellulose membranes. Protein levels of α smooth-muscle actin (αSMA, 1:500, A5228, Sigma-Aldrich, St. Louis, MO, United States), calsequestrin (CSQ, 1:2,500, PA1-913, Thermo Fisher Scientific), collagen 1α (Col1a, 1:1,000, sc-293182, Santa Cruz Biotechnology, Santa Cruz, CA, United States), connexin-40 (Cx40, 1:1,000, ab1726, Merck Millipore, Burlington, MA, United States), total and Ser368-phosphorylated connexin-43 (Cx43, 1:1,000, 3511, Cell Signaling Technology, Danvers, MA, United States), fibronectin (1:1,000, sc-8422, Santa Cruz Biotechnology), GAPDH (1:20,000, 5G4 6C5, HyTest, Turku, Finland), junctin (1:2,000, LS-C196703, LifeSpan BioSciences, Seattle, WA, United States), junctophilin-2 (1:1,000, sc-134875, Santa Cruz), matrix metallopeptidase 9 (MMP9, 1:200, ab38898, Abcam, Cambridge, United Kingdom), Na^+^-Ca^2+^-exchanger type-1 (NCX1, 1:1,000, R3F1, Swant, Marly, Switzerland), periostin (1:1,000, sc-134875, Santa Cruz Biotechnology), total, Ser16- and Thr17-phosphorylated phospholamban (PLB, all 1:1,000, ab2865 and ab92697, Abcam, Cambridge, United Kingdom and A010-13, Badrilla Ltd., Leeds, United Kingdom), total ryanodine receptor type-2 channel (RyR2, 1:1,000, MA3-916, Thermo Fisher Scientific), sarcolipin (1:100, ABT13, Merck Millipore), sarcoplasmic reticulum (SR) Ca^2+^-ATPase type-2a (SERCA2a; 1:2,000, sc-8095, Santa Cruz Biotechnology), transforming growth factor β1 (TGF-β1, 1:1000, ab9758, Abcam), and vimentin (1:1,000, sc373717, Santa Cruz Biotechnology) were determined using appropriate primary antibodies. The Ser2808-RyR2 and Ser2814-RyR2 phospho-epitope-specific antibodies (both 1:2,000) were custom generated, as previously described (Voigt et al., 2012). Appropriate near-infrared fluorophore dyes (IRDye, all 1:20,000, LI-COR Biosciences, Lincoln, NE, United States) were employed as secondary antibodies and imaged with an Odyssey Infrared Imaging System (LI-COR Biosciences). Protein expression was normalized to GAPDH.

### Electrophysiological Recordings

Action potentials were recorded in trabeculae isolated from RA appendages using the standard microelectrode technique as previously described, with microelectrode tip resistance of 10–25 MΩ and 1 ms stimulus, 25% above threshold intensity applied at a frequency of 1 Hz (Wettwer et al., 2004; Loose et al., 2014). The bath solution contained (in mmol/L): NaCl 127, KCl 4.5, MgCl_2_ 1.5, CaCl_2_ 1.8, glucose 10, NaHCO_3_ 22, and NaH_2_PO_4_ 0.42, equilibrated with O_2_/CO_2_ (95:5) at 35 ± 2°C and pH = 7.4.

Cardiomyocyte isolation was performed according to previously established protocols (Llach et al., 2011; Molina et al., 2012; Voigt et al., 2015). Briefly, enzymatic digestion was carried out in a Ca^2+^-free Tyrode solution containing 0.5 mg/ml collagenase (Worthington type 2, 300 u/mg), 0.25 mg/ml proteinase (Sigma type XXIV, 11 u/mg solid) and 5% bovine fatty acid-free albumin. After 30 min at 37°C, the tissue was removed from the enzyme solution, and cells were disaggregated in Ca^2+^-free solution. The remaining tissue was digested for 3 × 15 min in a fresh Ca^2+^-free solution containing 0.4 mg/ml collagenase. Only elongated cells with clear cross striations and without granulation were used for experiments. Total membrane currents were measured at room temperature in the perforated-patch configuration with an EPC-10 amplifier (HEKA Elektronik, Germany), as previously described (Llach et al., 2011; Molina et al., 2012). The extracellular solution contained (in mmol/L): NaCl 127, TEA 5, HEPES 10, NaHCO_3_ 4, NaH_2_PO_4_ 0.33, glucose 10, pyruvic acid 5, CaCl_2_ 2, MgCl_2_ 1.8 (pH = 7.4) and the pipette solution contained (in mmol/L): aspartic acid 109, CsCl 47, Mg_2_ATP 3, MgCl_2_ 1, Na_2_-phosphocreatine 5, Li_2_GTP 0.42, HEPES 10 (pH = 7.2 with CsOH). Amphotericin B (250 μg/ml) was added to the pipette solution before starting the experiment. All chemicals were acquired from Sigma-Aldrich.

The L-type calcium current (I_Ca,L_) was measured using a 50 ms prepulse from -80 to -45 mV to inactivate the fast Na^+^ current, followed by a 200 ms depolarization to +10 mV and I_Ca,L_ amplitude was determined as the difference between the peak inward current and the current at the end of the depolarization (Llach et al., 2011; Molina et al., 2012). The current–voltage relationship for I_Ca,L_ and the voltage-dependent inactivation were obtained using test potentials between -40 and +50 mV. The time constants of I_Ca,L_ inactivation were determined from a bi-exponential fit of the decaying phase of I_Ca,L_. Recovery of I_Ca,L_ from inactivation was assessed using a two-pulse protocol with increasing intervals between the first and the second pulse used to elicit I_Ca,L_.

The SR Ca^2+^ content was measured at room temperature as the time integral of the current elicited by rapid exposure to 10 mmol/L caffeine to release all Ca^2+^ from the SR, activating NCX1, and was converted to amoles (10^-18^ mol) of calcium released from the SR, assuming a stoichiometry of 3 Na^+^ to 1 Ca^2+^ for NCX1. Spontaneous calcium releases were examined as transient-inward currents (I_NCX_) with the membrane potential clamped at -80 mV, as previously described (Hove-Madsen et al., 2004; Llach et al., 2011; Molina et al., 2012; Voigt et al., 2012).

### RyR2 Single-Channel Recordings

RyR2 single-channel recordings were obtained as previously described (Wehrens et al., 2003). In brief, SR membrane-preparations were incorporated into lipid-bilayer membranes made of a 3:1 mixture of phosphatidylethanolamine and phosphatidylserine (Avanti Polar Lipids, Alabaster, AL, United States) dissolved in *n*-decane (25 mg/ml). Bilayers were formed across a 150 μm aperture of a polystyrene cuvette. The *trans* chamber (luminal side of the SR) contained (in mmol/L): HEPES 250, KCl 50 and Ca(OH)_2_ 53. The *cis* chamber (cytosolic side of the SR) contained (in mmol/L): HEPES 250, Tris-base 125, KCl 50, EGTA 1, CaCl_2_ 0.5 (pH = 7.35). At the end of each experiment, ryanodine (5 μmol/L) was applied to the *cis* chamber to confirm identity of RyR2 channels. Data were collected using Digidata 1322A (Molecular Devices, Sunnyvale, CA, United States) and Warner Bilayer Clamp Amplifier BC-535 (Warner Instruments, Hamden, CT, United States) at 0 mV under voltage-clamp conditions. Cytosolic free Ca^2+^ was calculated with WinMax32. Data were analyzed from digitized current recordings using pCLAMP-9.2 software (Molecular Devices).

### Data Analysis and Statistics

Data are presented as mean ± standard error (SEM) or scatter plots of individual measurements with 95% confidence interval and interquartile ranges. Normality was assessed using D’Agostino and Pearson omnibus test. For multicellular AP recordings, biochemical experiments, and clinical parameters, for which each patient contributed a single data point, one-way ANOVA followed by a *post hoc* Bonferroni *t*-test was used to compare means between groups for normally distributed continuous data. Continuous data which did not follow a normal distribution or for which normality could not be assessed were compared using a Kruskal–Wallis test with Dunn’s multiple comparison *t*-test. Categorical data were analyzed using a Chi-squared test with Bonferroni correction for multiple comparisons when comparing more than two groups. For patch-clamp and RyR2 single-channel recordings, in which each patient may contribute multiple data points, hierarchical statistics were employed according to recently published methods (Sikkel et al., 2017). Logarithmic transformations were applied to non-normal-distributed data (RyR2 properties and frequency of spontaneous SR Ca^2+^-release events) before applying hierarchical statistical analyses. *P* < 0.05 was considered statistically significant.

## Results

### Patient Characteristics

Major clinical parameters for all patient groups are provided in **Table 1** (for patient samples employed for multicellular AP recordings), **Table 2** (for patient samples used for cardiomyocyte isolation and voltage-clamp experiments), and **Table 3** (for patient samples used for biochemical experiments). By definition, LV ejection fractions were significantly lower in HFrEF and HFrEF-cAF groups compared to Ctl and cAF. In addition, there was a strong trend toward increased left atrial diameter in HFrEF patients, which was even more pronounced in cAF and HFrEF-cAF patients. As expected, HFrEF patients also tended to take more β-blockers, whereas AF patients more often received digitalis. There were no other major differences in clinical parameters between the three groups.

### Profibrotic Remodeling

**FIGURE 1:**
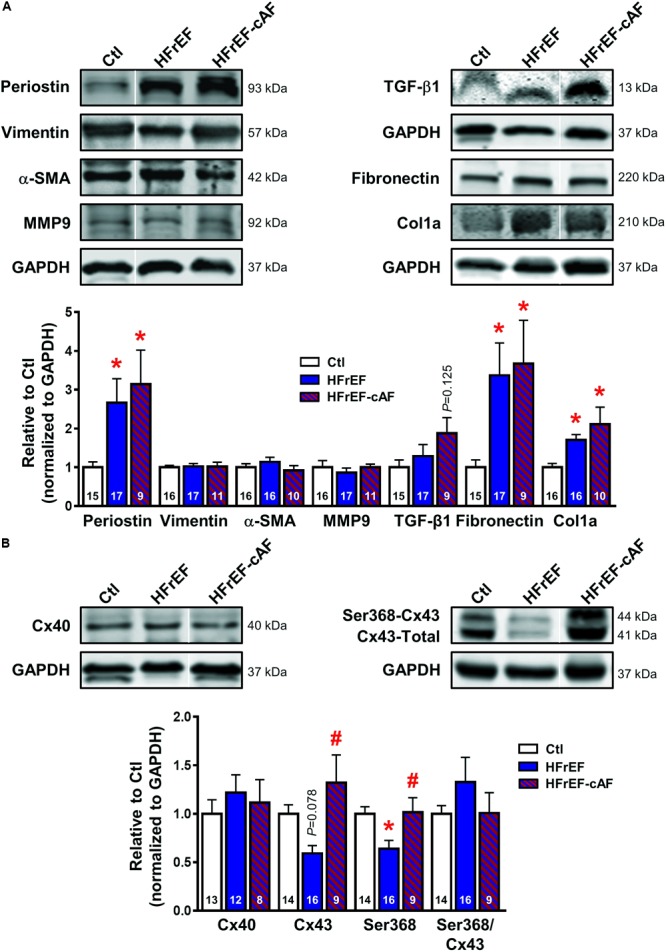
Atrial profibrotic and connexin remodeling. **(A)** Representative Western blot examples (top) and quantification of protein expression (bottom; mean ± SEM) of periostin, vimentin, α-smooth muscle actin (α-SMA), matrix metallopeptidase 9 (MMP9), transforming growth factor-β1 (TGF-β1), fibronectin and collagen 1α (col1a) in right-atrial tissue homogenates from Ctl (white bars), HFrEF (blue bars) or HFrEF-cAF (red/blue-striped bars) patients. Vertical white lines separate non-adjacent lanes on the same blot. **(B)** Representative Western blot examples (top) and quantification of protein expression (bottom; mean ± SEM) of total connexin-40 (Cx40), total and Ser368-phosphorylated connexin-43 (Cx43). GAPDH was used as loading control and is shown for the samples used for Western blots of periostin, vimentin, α-SMA and MMP9, for TGF-β1, and for fibronectin and col1a. Numbers in bars indicate number of patients. ^∗^ indicates *P* < 0.05 vs. Ctl, ^#^ indicates *P* < 0.05 vs. HFrEF.

Patients with HFrEF have increased macroscopic fibrosis and conduction slowing (Sanders et al., 2003; Akkaya et al., 2013). Moreover, animal models of HF consistently show increased molecular markers of profibrotic remodeling (Cardin et al., 2003). We evaluated protein expression levels of key extracellular matrix components (col1a, fibronectin, MMP9), activated fibroblast/(myo)fibroblast markers (vimentin, periostin, αSMA) and profibrotic signaling molecules (TGF-β1) in atrial tissue homogenates of Ctl, HFrEF and HFrEF-cAF patients (**Figure 1A**). Compared to Ctl patients, col1a, fibronectin and periostin were significantly increased in HFrEF and HFrEF-cAF patients, consistent with increased fibrosis in these patients.

### Electrical Remodeling

First we assessed the protein expression of connexin isoforms contributing to electrical cell-to-cell coupling and signaling. Protein levels of Cx40 were not different between the three groups, whereas protein expression of total Cx43 was reduced by ∼41% (*P* = 0.078) in atrial homogenates of HFrEF patients, but was not different between Ctl and HFrEF-cAF patients (**Figure 1B**). Similarly, steady-state phosphorylation levels of Cx43 at Ser368, which control internalization of connexins (Cone et al., 2014), were reduced by ∼36% in HFrEF only (**Figure 1B**), potentially contributing to the conduction slowing observed *in vivo* (Sanders et al., 2003).

**FIGURE 2:**
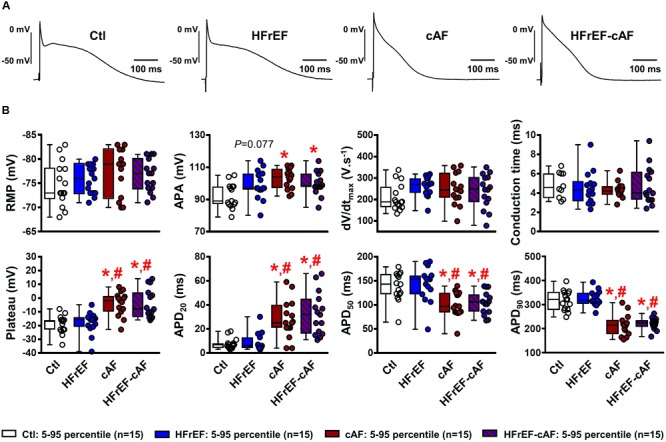
Electrical remodeling in multicellular human atrial trabeculae. **(A)** Representative action potential (AP) recordings in multicellular preparations from Ctl, HFrEF, cAF or HFrEF-cAF patients (from left to right). **(B)** Quantification of AP parameters in Ctl (white bars), HFrEF (blue bars), cAF (red bars) or HFrEF-cAF (red/blue-striped bars) patients. Top row shows (from left to right): resting membrane potential (RMP), AP amplitude (APA), maximal AP upstroke velocity (dV/dt_max_) and conduction time (note different n-numbers). Bottom row shows (from left to right): level of AP plateau and AP duration (APD) at 20%, 50%, and 90% of repolarization. ^∗^ indicates *P* < 0.05 vs. Ctl, ^#^ indicates *P* < 0.05 vs. HFrEF. Numbers indicate number of patients.

Next, we recorded APs in multicellular preparations from Ctl, HFrEF, cAF and HFrEF-cAF patients (**Figure 2A**). There were no significant differences in resting membrane potential, upstroke velocity (maximum dV/dt), conduction time, plateau potential or APD at 20%, 50%, or 90% of repolarization between Ctl and HFrEF patients (**Figure 2B**). By contrast, patients with cAF or HFrEF-cAF had a significantly increased plateau potential, significantly prolonged APD at 20% repolarization, and significantly shorter APD at 50% and 90% repolarization compared to Ctl (*P* < 0.01 for all; **Figure 2B**). There was a trend toward an increased AP amplitude in HFrEF (*P* = 0.077 vs. Ctl), which was significant in patients with cAF or HFrEF-cAF.

**FIGURE 3:**
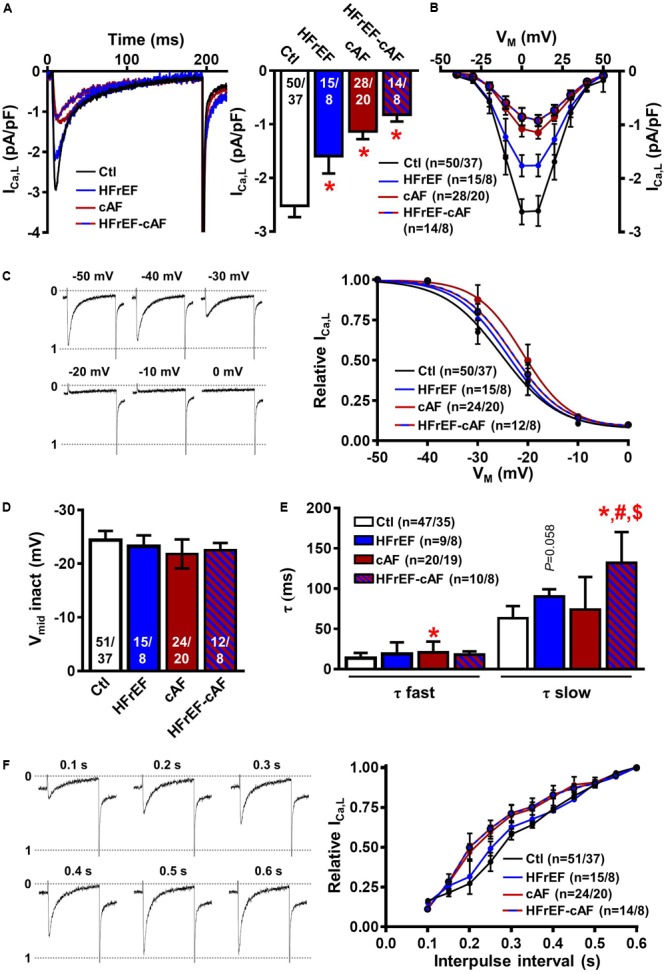
L-type Ca^2+^ current (I_Ca,L_) measurements in isolated human atrial cardiomyocytes. **(A)** Representative I_Ca,L_ recording during a 200-ms depolarizing pulse to +10 mV in a Ctl (black), HFrEF (blue), cAF (red) or HFrEF-cAF (blue/red) atrial cardiomyocyte (left) and quantification of I_CaL_ amplitude (right). **(B)** Current-voltage relationship of peak I_Ca,L_. **(C)** Representative examples of I_Ca,L_ inactivation protocol (left) and voltage dependence of inactivation (right) **(D)** midpoint of inactivation in Ctl, HFrEF, cAF and HFrEF-cAF patients. **(E)** Fast and slow time constants of inactivation during a depolarization to +10 mV in Ctl, HFrEF, cAF and HFrEF-cAF patients. **(F)** Representative examples (left) and quantification (right) of I_Ca,L_ recovery from inactivation with various interpulse intervals. Numbers in bars indicate number of cells/number of patients. ^∗^ indicates *P* < 0.05 vs. Ctl, ^#^ indicates *P* < 0.05 vs. HFrEF, ^$^ indicates *P* < 0.05 vs. cAF.

Then, we investigated I_Ca,L_ properties in isolated human atrial cardiomyocytes as a potential contributor to electrical remodeling and marker of Ca^2+^-handling remodeling. HFrEF, cAF and HFrEF-cAF patients all had a significant decrease in peak I_Ca,L_ amplitude (**Figure 3A**). Voltage-dependence of peak I_Ca,L_ (**Figure 3B**), inactivation (**Figures 3C,D**), and the time constant of recovery from inactivation (**Figure 3F**) were not different between Ctl, HFrEF, cAF and HFrEF-cAF patients. We determined the fast and slow time constants of I_Ca,L_ inactivation through a biexponential fit. Although the fast time constant was not different between Ctl, HFrEF and HFrEF-cAF (Ctl: 13.6 ± 0.95 ms, HFrEF: 18.8 ± 4.2 ms, and HFrEF-cAF: 18.1 ± 1.2 ms), it was increased in cAF (20.7 ± 2.9 ms). The slow time constant was significantly larger in HFrEF-cAF patients (131.8 ± 12.1 ms) and slightly increased in HFrEF (90.1 ± 3.2 ms; P = 0.058) compared to Ctl patients (63.2 ± 2.2 ms; **Figure 3E**). Taken together, these data point to potential remodeling of atrial Ca^2+^ handling in patients with HFrEF and HFrEF-cAF.

### Ca^2+^-Handling Remodeling

**FIGURE 4:**
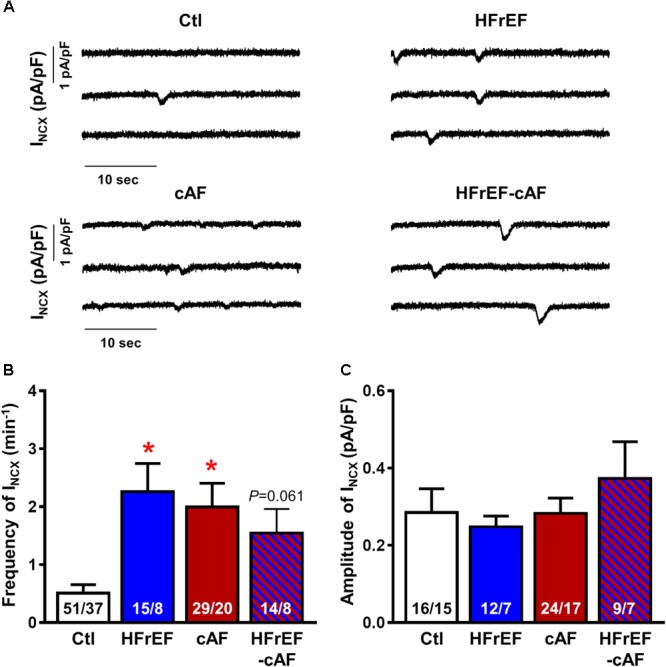
Spontaneous Ca^2+^-release events in isolated human atrial cardiomyocytes. **(A)** Representative recordings of NCX-mediated transient-inward current (I_NCX_) in atrial cardiomyocytes from Ctl, HFrEF, cAF or HFrEF-cAF patients. **(B,C)** Frequency **(B)** and amplitude **(C)** of spontaneous I_NCX_ in the four groups. Numbers in bars indicate number of cells/number of patients. ^∗^ indicates *P* < 0.05 vs. Ctl.

We first assessed indices of dysfunctional diastolic Ca^2+^ handling by measuring spontaneous occurrence of depolarizing transient-inward currents at -80 mV with perforated patch-clamp technique (**Figure 4A**), as described (Hove-Madsen et al., 2004). These currents, which are mediated by NCX, represent a well-accepted proarrhythmic consequence of spontaneous SR Ca^2+^-release events (Hove-Madsen et al., 2004; Llach et al., 2011; Heijman et al., 2014). The frequency of spontaneous I_NCX_ was larger in HFrEF (2.3 ± 0.49 min^-1^), cAF (2.0 ± 0.41 min^-1^), and HFrEF-cAF (1.5 ± 0.42 min^-1^) compared to Ctl (0.51 ± 0.14 min^-1^; **Figure 4B**), with unchanged I_NCX_ amplitude between the groups (**Figure 4C**).

**FIGURE 5:**
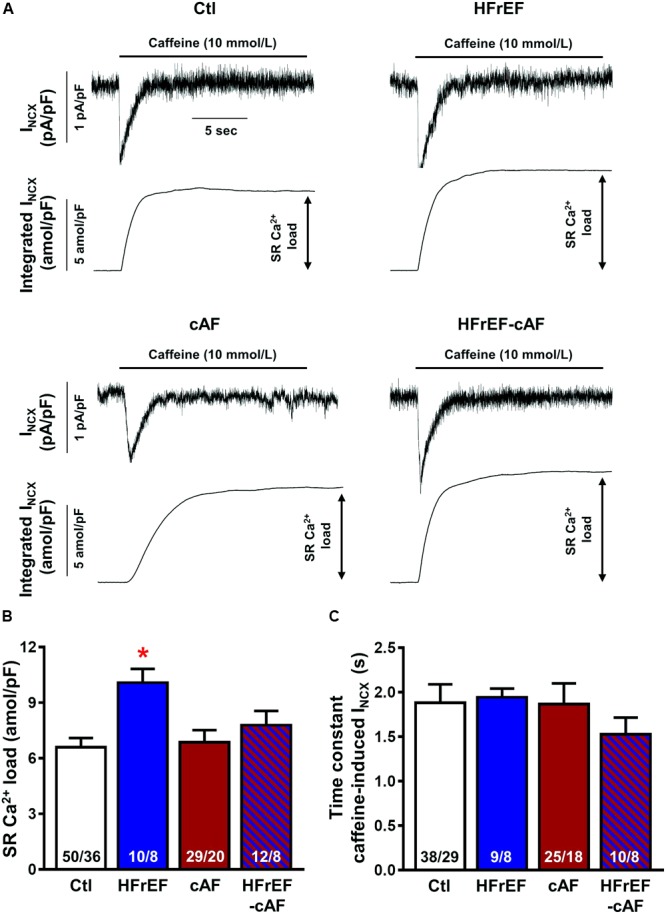
Sarcoplasmic reticulum (SR) Ca^2+^ load in isolated human atrial cardiomyocytes. **(A)** Representative recordings of NCX-mediated inward current (I_NCX_) induced by caffeine (10 mmol/l) application in atrial cardiomyocytes from Ctl, HFrEF, cAF or HFrEF-cAF patients (top panels). The bottom panels show the integral of membrane current over time, the amplitude of which is an accepted index of SR Ca^2+^ load. **(B,C)** SR Ca^2+^ load **(B)** and time constant of caffeine-induced I_NCX_ (an indicator of NCX function) in the four groups. Numbers in bars indicate number of cells/number of patients. ^∗^ indicates *P* < 0.05 vs. Ctl.

**FIGURE 6:**
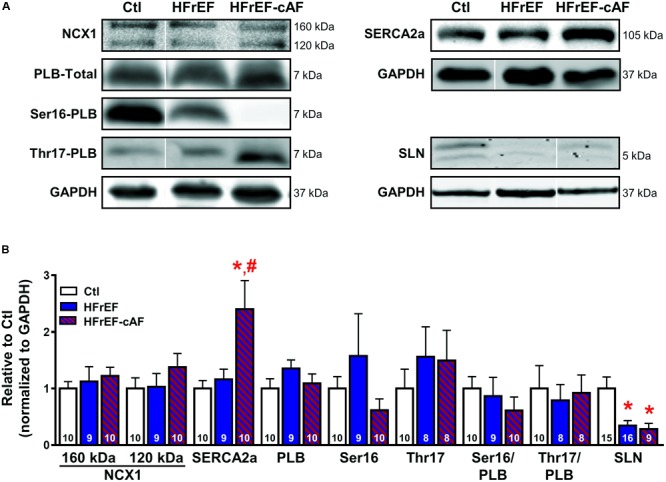
Molecular determinants of increased sarcoplasmic reticulum (SR) Ca^2+^ load. **(A)** Representative Western blot examples of protein expression of Na^+^-Ca^2+^-exchanger type-1 (NCX1; full-length protein of 160 kDa with proteolytic fragment at 120 kDa), SR Ca^2+^-ATPase type-2a (SERCA2a) and its inhibitory regulators phospholamban (PLB; total PLB as well as Ser16- and Thr17-phosphorylated PLB) and sarcolipin (SLN). GAPDH was used as loading control. Ser16-PLB of HFrEF-cAF represents an extreme example. **(B)** Quantification of NCX1, SERCA2a, total PLB, Ser16- and Thr17-phosphorylated PLB, Ser16/total PLB, Thr17/total PLB, and SLN in right-atrial tissue homogenates of Ctl (white bars), HFrEF (blue bars) or HFrEF-cAF (red/blue-striped bars). Numbers in bars indicate number of patients. ^∗^ indicates *P* < 0.05 vs. Ctl, ^#^ indicates *P* < 0.05 vs. HFrEF.

Spontaneous SR Ca^2+^-release events could be promoted by an increased SR Ca^2+^ load. Therefore, we quantified SR Ca^2+^ load during caffeine-mediated SR Ca^2+^ release, which produces a large I_NCX_, the integral of which is a reliable measure of SR Ca^2+^ content (Hove-Madsen et al., 2004; Voigt et al., 2012, 2014). SR Ca^2+^ load was significantly larger in HFrEF (10.1 ± 0.75 amol/pF) compared to Ctl (6.6 ± 0.50 amol/pF; *P* < 0.01; **Figures 5A,B**), but unchanged in cAF or HFrEF-cAF. The time constant of the caffeine-induced I_NCX_ was not different between the groups, pointing to an unaltered NCX function (**Figure 5C**). Accordingly, NCX1 protein expression was unchanged in HFrEF and HFrEF-cAF patients (**Figure 6**).

The increased SR Ca^2+^ load could be mediated by increased SR Ca^2+^ uptake via SERCA2a. Protein levels of SERCA2a were unchanged in HFrEF, but strongly increased in patients with HFrEF-cAF (**Figure 6**). Protein expression of the inhibitory SERCA2a regulator PLB, along with phosphorylation of PLB at the protein kinase-A site (Ser16) or the Ca^2+^/calmodulin-dependent protein kinase-II site (Thr17) were not different between Ctl, HFrEF and HFrEF-cAF patients (**Figure 6**). By contrast, expression of the atrial-specific SERCA2a inhibitor sarcolipin was decreased by ∼66% in HFrEF and by ∼72% in HFrEF-cAF (*P* < 0.05 vs. Ctl for both), likely increasing SR Ca^2+^ uptake.

**FIGURE 7:**
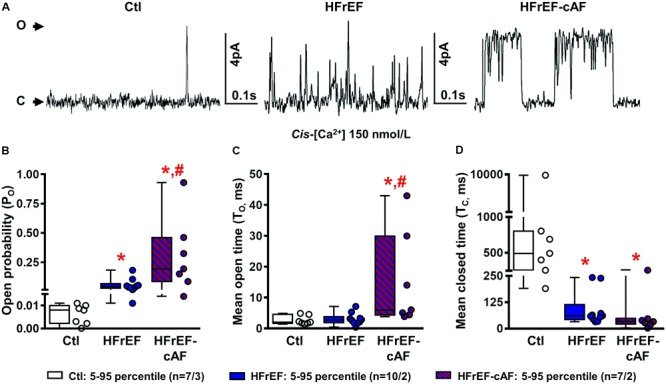
Single-channel recordings of cardiac ryanodine receptor type-2 (RyR2) channels. **(A)** Representative RyR2 single-channel recording in a right-atrial sample from a Ctl, HFrEF or HFrEF-cAF patient. Upward deflections reflect channel opening. **(B–D)** Quantification of open probability **(B)**, mean open time **(C)**, and mean closed time **(D)** of single RyR2 channels from Ctl (white bars/symbols), HFrEF (blue bars/symbols) or HFrEF-cAF (red/blue-striped bars/symbols) patients. Numbers represent number of channels/number of patients. ^∗^ indicates *P* < 0.05 vs. Ctl, ^#^ indicates *P* < 0.05 vs. HFrEF.

**FIGURE 8:**
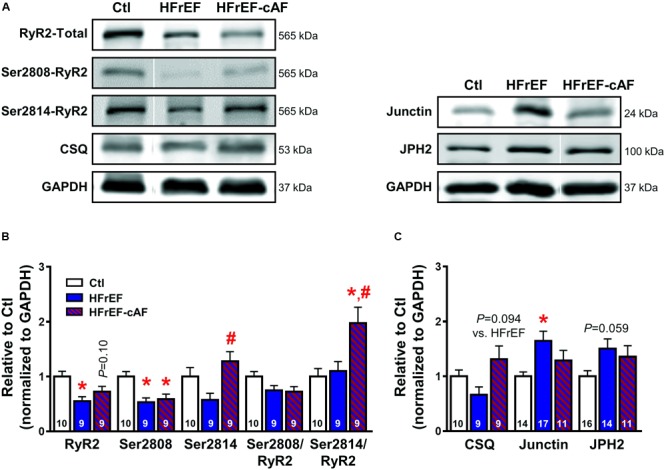
Molecular determinants of ryanodine receptor type-2 (RyR2) dysfunction. **(A)** Representative Western blot examples of total RyR2, Ser2808-, and Ser2814-phosphorylated RyR2 and calsequestrin (CSQ; left), as well as junctin and junctophilin-2 (JPH2; right) protein expression levels. GAPDH was used as loading control. **(B)** Quantification of total RyR2, Ser2808-, and Ser2814-phosphorylated RyR2, Ser2808/total RyR2, and Ser2814/total RyR2 protein expression levels in Ctl (white bars), HFrEF (blue bars) and HFrEF-cAF (red/blue-striped bars) patients. **(C)** Protein expression levels of the RyR2-interacting proteins CSQ, junctin, and JPH2 in Ctl, HFrEF and HFrEF-cAF patients. Data are shown relative to Ctl. Numbers in bars indicate number of patients. ^∗^ indicates *P* < 0.05 vs. Ctl, ^#^ indicates *P* < 0.05 vs. HFrEF.

Besides increased SR Ca^2+^ load, spontaneous SR Ca^2+^-release events often result from RyR2 dysfunction (Voigt et al., 2012, 2014; Beavers et al., 2013; Li et al., 2014). To address this directly we investigated RyR2 single-channel open probability in lipid bilayer experiments (**Figure 7A**). At 150 nmol/L intracellular [Ca^2+^], mimicking diastolic conditions, RyR2 open probability was significantly larger in both HFrEF (5.7 ± 1.6%) and HFrEF-cAF (30.9 ± 11.8%) patients compared to Ctl (0.6 ± 0.2%) patients (*P* < 0.05 for both; **Figure 7B**). The increased open probability was primarily due to a decrease in closed time (**Figure 7D**), although single-channel open time was significantly prolonged in HFrEF-cAF patients (**Figure 7C**), further increasing open probability. Finally, we assessed the molecular basis of the increased RyR2 open probability using Western blotting (**Figure 8A**). Compared to Ctl the relative RyR2 protein levels were decreased by ∼45% in HFrEF patients (*P* < 0.01), along with a similar non-significant trend (∼28% reduction) in HFrEF-cAF (*P* = 0.10; **Figure 8B**). Phosphorylation levels at Ser2808-RyR2 normalized to total RyR2 were not different between the three groups, whereas relative phosphorylation at Ser2814-RyR2 was increased by ∼97% in HFrEF-cAF compared to Ctl or HFrEF patients (*P* < 0.05 for both), potentially underlying the increased RyR2 open probability in these patients (**Figure 8B**). We also investigated the protein levels of other validated components of the RyR2 macromolecular complex (CSQ, junctin, and junctophilin-2) (Landstrom et al., 2017). Protein levels of CSQ were unchanged in HFrEF or HFrEF-cAF compared to Ctl (**Figure 8C**), whereas junctin expression was ∼65% higher in HFrEF vs Ctl patients (*P* = 0.011) and there was a trend toward increased levels of junctophilin-2 in HFrEF compared to Ctl patients (*P* = 0.059; **Figure 8C**).

## Discussion

In the present study, we have detailed for the first time profibrotic, electrical and Ca^2+^-handling remodeling in patients with HFrEF alone and HFrEF-cAF. Our data show (1) that profibrotic markers are upregulated in patients with HFrEF, independent of the presence of AF; (2) that protein expression and phosphorylation of connexin-43 are reduced in HFrEF only; (3) that classical indices of AF-related atrial electrical remodeling only occur in cAF and HFrEF-cAF; and (4) that potentially proarrhythmic atrial Ca^2+^-handling abnormalities are a typical finding in both HFrEF and HFrEF-cAF patients. Together, these data provide novel insights into the cellular and molecular mechanisms of AF in patients with HFrEF and HFrEF-cAF, which may have important implications for the development of novel therapeutic option for AF patients in the context of HF.

### Comparison to Previous Work

Atrial structural remodeling is a consistent finding in patients with HFrEF. At the tissue level, increased atrial fibrosis assessed using late gadolinium enhancement magnetic resonance imaging has been reported (Akkaya et al., 2013). Atrial fibrosis is also a common finding in animal models of HFrEF (Li et al., 1999; Milliez et al., 2005; Yamada et al., 2017; Pluteanu et al., 2018), and the underlying pathways have been investigated in detail (Cardin et al., 2003). Our data similarly point to prominent profibrotic remodeling, with increased expression of col1a, periostin and fibronectin. The associated alterations in atrial structure are expected to cause reentry-promoting slow, heterogeneous conduction, which has been directly observed *in vivo* in atria of HFrEF patients (Sanders et al., 2003). We also identified reduced expression and Ser368 phosphorylation of Cx43 in HFrEF patients, which may also contribute to conduction abnormalities.

Reentry-promoting electrical remodeling, characterized by shortening of APD and hyperpolarization of the resting membrane potential, is a hallmark of cAF and is a consistent finding in animal models with atrial tachycardia remodeling (Heijman et al., 2014). By contrast, APD and resting membrane potential of patients with paroxysmal AF are not different from Ctl patients (Voigt et al., 2014; Schmidt et al., 2015). Similarly, in the present study we found no significant differences in AP properties in patients with HFrEF only, suggesting that electrical remodeling may be primarily a consequence of the rapid atrial rate during cAF. At the cardiomyocyte level various inconsistent results have been published about atrial electrical remodeling in the setting of HF. Workman et al. (2009) reported shortened APD and effective refractory period (ERP) in atrial cardiomyocytes from HFrEF patients undergoing cardiac surgery, whereas Schreieck et al. (2000) detected no differences in atrial cardiomyocyte APD between HFrEF and Ctl patients or healthy donors. Finally, other studies in atrial cardiomyocytes (Koumi et al., 1994), *ex vivo* multicellular preparations (Fedorov et al., 2011) and *in vivo* recordings in patients (Sanders et al., 2003) have reported prolonged APD and/or ERP.

The inconsistent results between studies related to APD in HFrEF patients might at least in part be explained by the use of isolated cardiomyocytes vs. multicellular preparations and the techniques used to assess APD/ERP. Cardiomyocyte isolation procedures from human atria are known to affect function of a number of ion channels, often resulting in depolarized resting membrane potentials that require injection of a hyperpolarizing current in order to record APs. Such a current may differentially affect APs from Ctl or HFrEF patients. In addition, remodeling of individual ion currents in HFrEF patients appears highly variable between studies. For example, both reduced and unchanged inward-rectifier K^+^ current, as well as reduced, unchanged and increased transient-outward K^+^ current have been reported (Koumi et al., 1994; Van Wagoner et al., 1997; Schreieck et al., 2000; Workman et al., 2009). Differences in patient characteristics, including the use of explanted end-stage failing hearts and healthy donor hearts in some studies (Koumi et al., 1994; Van Wagoner et al., 1997; Fedorov et al., 2011), compared to patients undergoing open-heart surgery with normal or impaired LV function in our and other studies (Schreieck et al., 2000; Workman et al., 2009), may also contribute to the variable findings. Similarly, work from animal models suggest that the duration of HF differentially affects electrical remodeling, with 2 weeks of ventricular tachypacing resulting in ERP prolongation (Cha et al., 2004), 5 weeks of ventricular tachypacing associating with unchanged ERP (Li et al., 1999) and 4 months of ventricular tachypacing abbreviating atrial ERP (Sridhar et al., 2009). Since the duration of HFrEF is usually not controlled in patients, differences in HFrEF duration between the human studies are expected. Here we employed sharp-electrode AP recordings in multicellular preparations, which avoided cell-isolation-induced effects, minimized the variability in individual ion currents and produced stable resting membrane potentials at physiological levels. Our results suggest that even if remodeling in some ion currents might exist in HFrEF patients, resting membrane potential and APD shape do not differ between Ctl and HFrEF patients. Clearly, validation of our multicellular findings in other patient cohorts is needed to confirm whether alterations in atrial APD occur with HFrEF.

Our current data indicate that also in the presence of HFrEF, AF produces typical atrial tachycardia-related remodeling, including reentry-promoting APD shortening. These data are consistent with results from Cha et al. who showed that atrial tachycardia shortened ERP in dogs with pacing-induced HF, albeit to a lesser degree than dogs with normal LV function (Cha et al., 2004). By contrast, in a pig model the combined presence of AF and HF resulted in prolonged APD (Lugenbiel et al., 2015). However, in this model AF was the primary initiator of a (mild) ventricular tachycardiomyopathy, which is different from patients in whom HF is mainly due to advanced cardiovascular disease, notably hypertension and myocardial infarction (Benjamin et al., 2018), as in our study. Overall, despite the fact that potential APD changes can occur with HFrEF, once AF develops it produces the APD shortening that typifies the electrophysiological profile in patients with cAF.

To the best of our knowledge, functional Ca^2+^-handling remodeling has not been studied in atria of patients with HFrEF. Here we identified extensive indices of abnormal Ca^2+^-handling in these patients, both in the absence and presence of AF, including greater incidence of spontaneous I_NCX_, increased SR Ca^2+^ load, and RyR2 dysfunction. Protein levels of sarcolipin and RyR2 were decreased in HFrEF patients, but we did not find significant changes in SERCA2a or NCX1 expression, PLB expression or phosphorylation, or RyR2 phosphorylation in these patients. Our results are consistent with data from Shanmugam et al. (2011) who also identified decreased sarcolipin expression, unchanged SERCA2a expression and a tendency toward decreased RyR2 expression in atria from HF patients. The present results are also in line with data in atrial cardiomyocytes from rabbits with chronic myocardial infarction (Kettlewell et al., 2013), failing hearts from spontaneously hypertensive rats (Pluteanu et al., 2018), and a dog model of ventricular tachypacing-induced HF (Stambler et al., 2003; Yeh et al., 2008), all of which showed an increased incidence of spontaneous SR Ca^2+^-release events. Moreover, HF dogs also had increased SR Ca^2+^ load, decreased RyR2 expression, unaltered RyR2 phosphorylation and unchanged expression of PLB or NCX1 (Yeh et al., 2008), similar to our data. In contrast to our findings, SERCA2a expression was decreased in this model and the significantly increased SR Ca^2+^ load was explained by an increase in PLB phosphorylation at Thr17 (Yeh et al., 2008). Finally, our data in HFrEF patients are similar to our previous findings in patients with paroxysmal AF (Voigt et al., 2014). Paroxysmal AF patients also exhibited increased SR Ca^2+^ load and spontaneous SR Ca^2+^-release events, although the underlying molecular mechanisms appear somewhat distinct, involving increased RyR2 expression and decreased SERCA2a expression. Interestingly, despite a similar functional phenotype, there also appear to be mechanistic differences at the molecular level between HFrEF and HFrEF-cAF patients. For example, although protein levels of sarcolipin and RyR2 are decreased in both HFrEF and HFrEF-cAF patients, only the latter group have upregulated SERCA2a expression along with increased RyR2-Ser2814 phosphorylation, which sensitizes RyR2 to cytosolic Ca^2+^. The higher RyR2-Ser2814 phosphorylation level is consistent with the significantly greater RyR2 single-channel open probability in HFrEF-cAF compared to Ctl or HFrEF patients, likely explaining why SR Ca^2+^ load is not increased in HFrEF-cAF. The increased SERCA2a expression might also help to maintain SR Ca^2+^ load despite the strong increase in RyR2 open probability, which is expected to increase SR Ca^2+^ leak and to decrease SR Ca^2+^ load. Finally, protein levels of junctin were higher in HFrEF, but not HFrEF-cAF patients and mice with cardiac restricted junctin overexpression develop spontaneous AF (Hong et al., 2002). Thus junctin-mediated RyR2 dysfunction might contribute to the development of AF in the context of HF.

### Clinical Implications

Despite the important progress being made in our understanding, diagnosis and treatment of AF, it remains a major clinical problem (Heijman et al., 2018). Basic science has provided a lot of insights about the fundamental mechanisms underlying AF, identifying ectopic (triggered) activity promoted by Ca^2+^-handling abnormalities and reentry promoted by APD shortening and slow, heterogeneous conduction as the major arrhythmia mechanisms (Heijman et al., 2014, 2016). Nonetheless, a number of important translational challenges remain (Heijman et al., 2018), including the identification of the dominant arrhythmia mechanisms in specific subgroups of AF patients to enable tailored therapy. There is increasing awareness that AF is a common endpoint of many different pathophysiological processes that promote an atrial cardiomyopathy (Andrade et al., 2014; Goette et al., 2017). However, the nature of the underlying atrial remodeling is likely distinct for different comorbidities, risk factors and stages of the disease. In agreement, we have previously shown that there are important differences in atrial remodeling between patients with paroxysmal AF and those with cAF (Voigt et al., 2012, 2014; Beavers et al., 2013). Atrial remodeling in patients with HFrEF or HFrEF-cAF has not previously been characterized in detail, particularly with respect to atrial Ca^2+^-handling, despite the very common clinical coexistence of AF and HF (Ling et al., 2016; Sartipy et al., 2017).

Together with previous studies, our current data suggest that Ca^2+^-handling abnormalities and Ca^2+^-mediated triggered activity are a common motif shared by cAF patients and HF patients in sinus rhythm, who are at an increased risk of developing AF. Atrial arrhythmias have a focal initiation pattern in dogs with ventricular tachypacing-induced HF (Fenelon et al., 2003) and their inducibility is suppressed by Ca^2+^-antagonists (Stambler et al., 2003), supporting a major role for Ca^2+^-mediated ectopic activity in the initiation of AF in the setting of HF. In addition, there is evidence for a significant role of focal ectopy in AF patients (Haissaguerre et al., 1998; Lee et al., 2015).

Accumulating evidence suggests that Ca^2+^-handling abnormalities may promote structural remodeling. For example, CREM-IbΔC-X transgenic mice develop progressive atrial remodeling and spontaneous AF, which is prevented by genetic inhibition of RyR2 dysfunction and associated SR Ca^2+^ leak (Li et al., 2014). Similarly, the PLB-R14del mutation produces Ca^2+^-handling abnormalities and exhibits a clinical phenotype characterized initially by an increased risk of ventricular arrhythmias at young age, with a subsequent predisposition to dilated cardiomyopathy and HF (van Rijsingen et al., 2014). Thus, abnormal Ca^2+^-handling might be an early event in the pathogenesis of AF, apart from promotion of ectopic activity *per se*. In particular, the HFrEF-induced atrial Ca^2+^-handling remodeling that we identified may serve both as an initiator of AF episodes and a promoter of atrial electrical and structural remodeling, creating a substrate for the maintenance and progression of AF. Thus, our present findings may have important clinical implications. They point to an urgent need for methods to assess potential cardiomyocyte Ca^2+^-handling abnormalities in patients during the early stages of the remodeling process, when the disease is still amenable to therapy, and a need for better therapeutic options to treat these abnormalities based on our understanding of the underlying molecular mechanisms in specific patient subgroups (Heijman et al., 2018). Moreover, since atrial Ca^2+^-handling remodeling in HFrEF patients is characterized by both RyR2 and SERCA2a dysfunction, a therapeutic intervention modulating only a single target may not be sufficient to normalize altered atrial Ca^2+^ handling. Indeed, we have previously shown in paroxysmal AF patients with a similar phenotype that RyR2 and SERCA2a dysfunction can each promote proarrhythmic spontaneous SR Ca^2+^-release events and that the combination of both factors is significantly more proarrhythmic than each individually (Voigt et al., 2014). Technological advances such as catheter-based photoacoustic imaging with cell-permeable Ca^2+^ indicators (Roberts et al., 2018) may in the future enable *in vivo* imaging of Ca^2+^-handling abnormalities in large animal models or patients (Heijman et al., 2018).

### Potential Limitations

Although our data provide an extensive characterization of atrial remodeling in HFrEF and HFrEF-cAF patients, we did not address ion-channel remodeling in detail. Despite absence of AP changes in multicellular preparations, I_Ca,L_ was reduced in HFrEF patients, in line with previous findings (Ouadid et al., 1995). Thus, alterations in K^+^ channels may balance the reduction in I_Ca,L_, resulting in unaltered AP properties, as previously observed in other studies (Koumi et al., 1994; Schreieck et al., 2000; Workman et al., 2009).

We did not measure Ca^2+^-handling abnormalities directly using Ca^2+^ imaging with fluorescent indicators. Instead, we employed spontaneous I_NCX_ during voltage-clamp at -80 mV as an accepted marker of spontaneous SR-derived Ca^2+^ release and we characterized Ca^2+^-handling remodeling through changes in RyR2 single-channel properties. Of note, the use of perforated-patch techniques with preserved physiological intracellular milieu, along with the absence of fluorescent Ca^2+^ indicators, which buffer intracellular Ca^2+^, are expected to result in a more precise detection of the consequences of abnormal SR Ca^2+^ releases. Nevertheless, subsequent work employing Ca^2+^ imaging should further dissect the precise abnormalities of atrial Ca^2+^-handling in the context of HFrEF with and without AF.

The present work is necessarily restricted to RA appendage tissue obtained from patients undergoing open-heart surgery. Our findings may not hold for other regions of the atria, which may be exposed to different levels of stretch or neurohumoral factors, and may not be applicable to HFrEF patients without an indication for open-heart surgery.

We have previously shown that there are important differences in Ca^2+^-handling abnormalities between paroxysmal AF and cAF (Voigt et al., 2012, 2014). Similarly, there are notable differences in the epidemiology, pathophysiology and therapeutic management of HF patients with reduced, mid-range and preserved LV ejection fraction, although AF is common in all variants (Lam and Solomon, 2014; Sartipy et al., 2017). Our results were obtained in patients with significantly impaired LV function (mean LV ejection fractions ∼30%) with or without cAF. Although the clinical characteristics of the HFrEF population employed in this population make an ischemic etiology very likely, we were unable to establish the exact etiology of HF for individual patients. Results might be different for other types of AF and HF, which could produce distinct forms of atrial remodeling and show complex patterns in their interaction. Furthermore, there were some differences in clinical characteristics (e.g., regarding the indication for surgery) among the patient groups and the different data sets for the same subgroup (i.e., between **Tables 1–3**), which might influence our findings. Finally, our study is concerned with the potential molecular and cellular mechanisms of AF in HFrEF patients, but although we were able to include data from cAF without HFrEF in our functional analyses, we did not have samples from cAF patients without HFrEF to include in the Western-blot experiments. Furthermore, our work does not address the additional tissue-level properties, such as fibrosis and conduction velocity patterns or autonomic innervation, which play a key role in cardiac arrhythmogenesis in patients. For example, we did not observe significant differences in maximum dV/dt_max_ (reflecting Na^+^-channel function) and conduction time, which also reflects connexin function, in multicellular preparations of HFrEF patients, despite reduced Cx43 protein levels and reports of reduced conduction velocity *in vivo* (Sanders et al., 2003), suggesting a role for regional differences and additional regulatory mechanisms of atrial conduction in the intact heart.

## Conclusion

In this study, we provide the first detailed characterization of atrial remodeling in the setting of HFrEF with and without cAF. Spontaneous I_NCX_ currents, likely mediated by Ca^2+^-handling abnormalities, are increased and might act as triggers of AF in HFrEF patients with profibrotic and connexin remodeling. AF subsequently produces atrial electrical remodeling that stabilizes the arrhythmia by promoting AF-maintaining reentry. These novel findings may have important implications for the development of new treatment options for AF in the context of HF.

## Author Contributions

CM and DD conceived the research. CM, IA-T, and QW performed the experiments. ER-D and MK provided reagents and samples. CM, IA-T, QW, SN, UR, XW, JH, and DD analyzed the data. XW, LH-M, and DD handled funding and project management. JH, SN, and DD drafted and revised the manuscript. All authors approved the final version of the manuscript.

## Conflict of Interest Statement

XW is a founding partner of Elex Biotech, a start-up company that developed drug molecules that target ryanodine receptors for the treatment of cardiac arrhythmia disorders. DD is a member of the scientific advisory board of OMEICOS Therapeutics GmbH, a company developing small molecules mimicking the effects of omega-3 fatty acids, and of Acesion Pharma, a company developing selective blockers of small-conductance calcium dependent potassium channels. The remaining authors declare that the research was conducted in the absence of any commercial or financial relationships that could be construed as a potential conflict of interest.

## Abbreviations

AF: atrial fibrillation
AP: action potential
APD: AP duration
αSMA: α smooth muscle actin
cAF: long-standing persistent (‘chronic’) AF
Col1a: collagen 1a
CSQ: calsequestrin
Cx40/43: connexin-40/connexin-43
ERP: effective refractory period
HF: heart failure
HFrEF: heart failure with reduced ejection fraction
I_Ca,L_: L-type Ca^2+^ current
I_TI_: transient-inward current
LV: left ventricle
MMP9: matrix metallopeptidase 9
NCX1: Na^+^-Ca^2+^-exchanger type-1
NYHA: New York Heart Association
PLB: phospholamban
RA: right atrium
RyR2: ryanodine receptor type-2
SERCA2a: sarcoplasmic reticulum Ca^2+^-ATPase type-2a
SR: sarcoplasmic reticulum
TGF-β1: transforming growth factor β1
